# INTERGENERATIONAL CONVERSATIONS AS GOOD AGE-FRIENDLY UNIVERSITY PRACTICE

**DOI:** 10.1093/geroni/igac059.1410

**Published:** 2022-12-20

**Authors:** Cynthia Hancock, Katie Kutcher

**Affiliations:** UNC Charlotte, Charlotte, North Carolina, United States; UNC Charlotte, Charlotte, North Carolina, United States

## Abstract

Service-learning is an established practice in gerontological education. At UNC Charlotte, our service-learning is embedded in our foundational gerontology course which is open to students across the University. Students engage in 10 hours of conversations utilizing topic prompts that align with course content to engage in comfortable conversations with an older adult they have never before met. Students then complete five reflective writings to process the experience. Our model aligns with the AFU principles of promoting “intergenerational learning” and increasing “the understanding of students of the longevity dividend and the increasing complexity and richness that aging brings to our society.” Through a qualitative analysis of student reflective writings, the authors will share how this service-learning model leads to increased student gerontological literacy and in particular a better understanding of their future aging selves.

